# Supplementary material for: Genetic Dissection of Drought Tolerance in Maize Through GWAS of Agronomic Traits, Stress Tolerance Indices, and Phenotypic Plasticity
Source: Int J Mol Sci. 2025 Jun 29;26(13):6285. doi: 10.3390/ijms26136285 (PMC12249902; doi:10.3390/ijms26136285)
Supplement: Supplementary file 1 [file ijms-26-06285-s001.zip › Supplementary figures.pdf]

Supplementary Figures

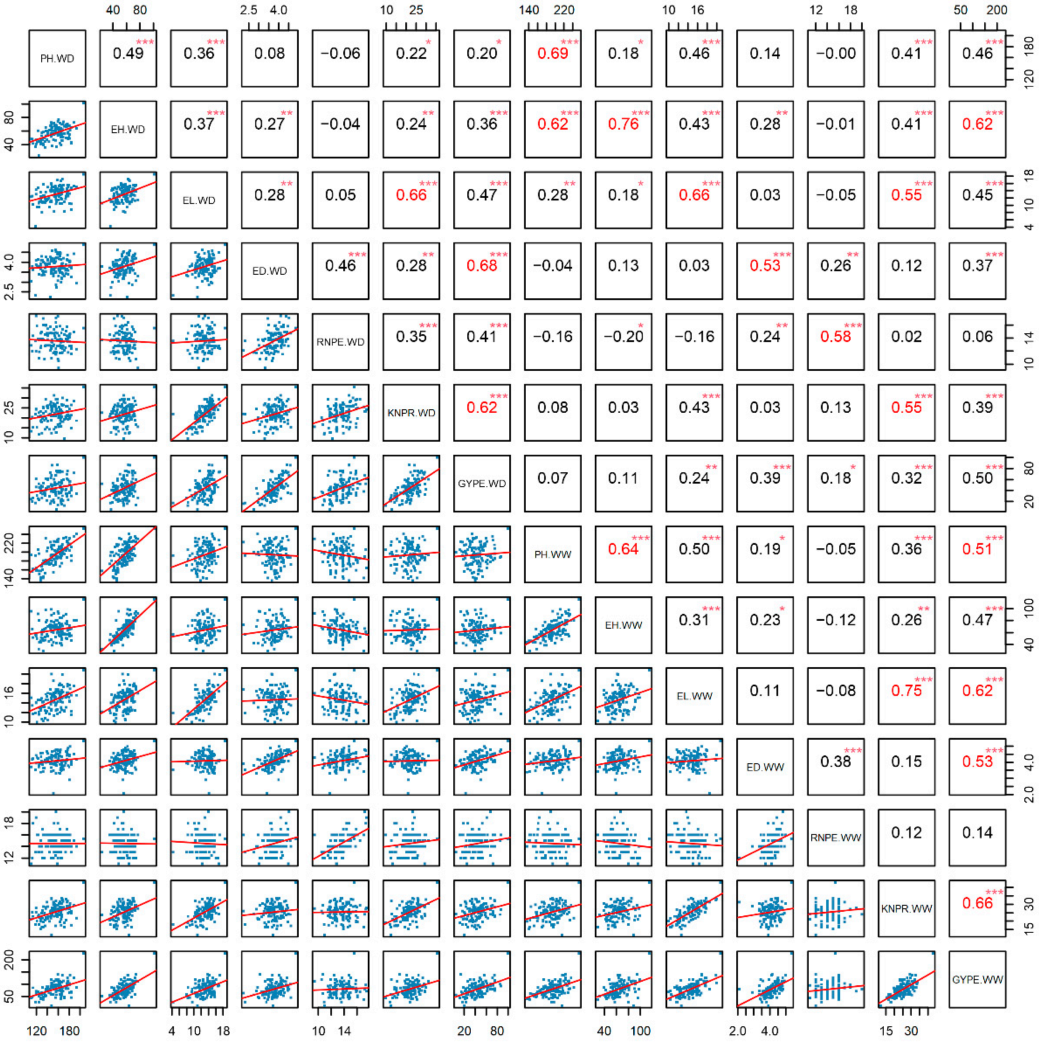

Figure S1. Correlations of seven agronomic traits under well-watered (WW) and water-deficit (WD) in Urumqi. Note: *:P < 0.05; **:P < 0.01, ***:P < 0.001. In the upper right corner, the numbers of red or deep red indicate moderate or high correlation, respectively. The red lines represent the trend of correlation in the lower left corner. PH: plant height, EH: ear height, GYPE: grain yield per ear, EL: ear length, ED: ear diameter, RNPE: row number per ear, KNPR: kernel number per row.

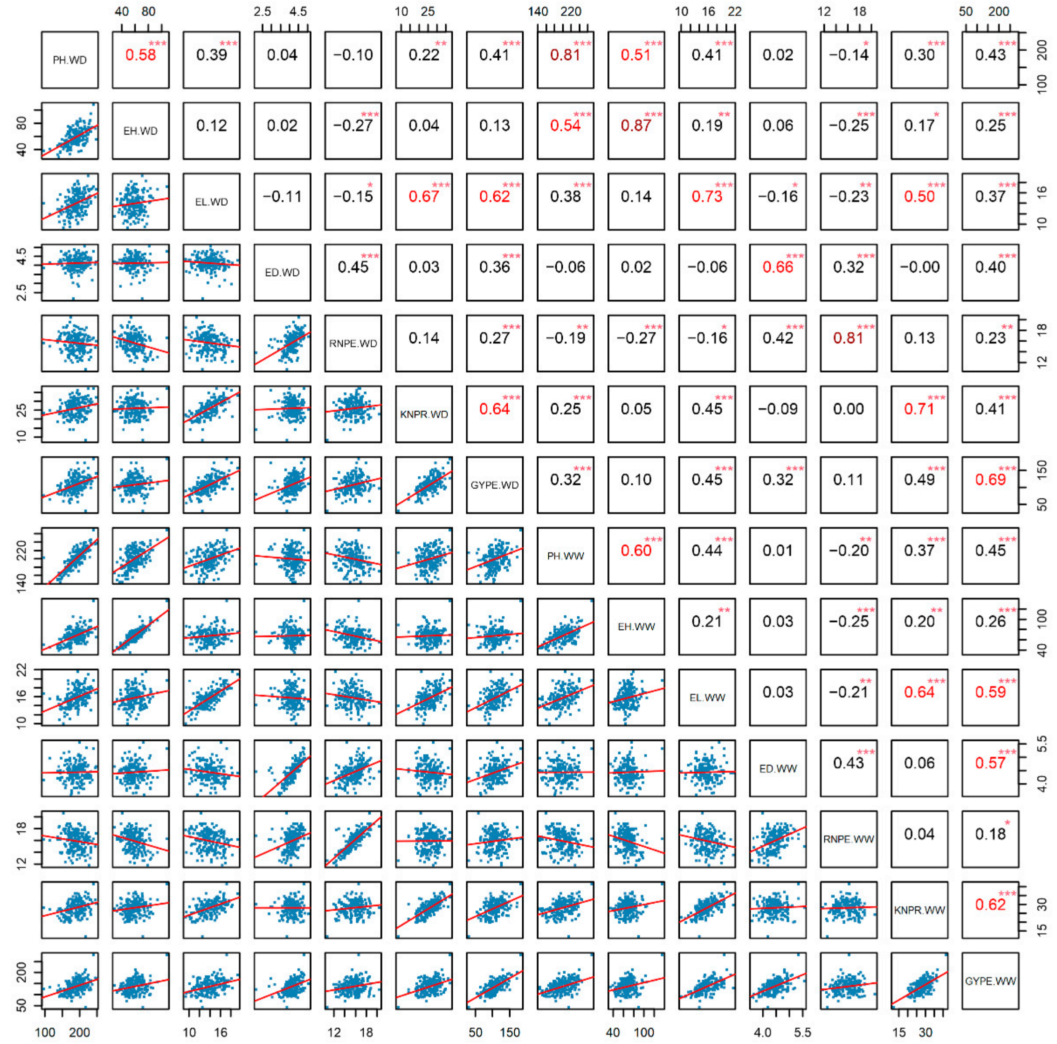

Figure S2. Correlations of seven agronomic traits under well-watered (WW) and water-deficit (WD) in Yulin. Note: *:$P < 0.05$; **:$P < 0.01$, ***:$P < 0.001$. In the upper right corner, the numbers of red or deep red indicate moderate or high correlation, respectively. The red lines represent the trend of correlation in the lower left corner. PH: plant height, EH: ear height, GYPE: grain yield per ear, EL: ear length, ED: ear diameter, RNPE: row number per ear, KNPR: kernel number per row.

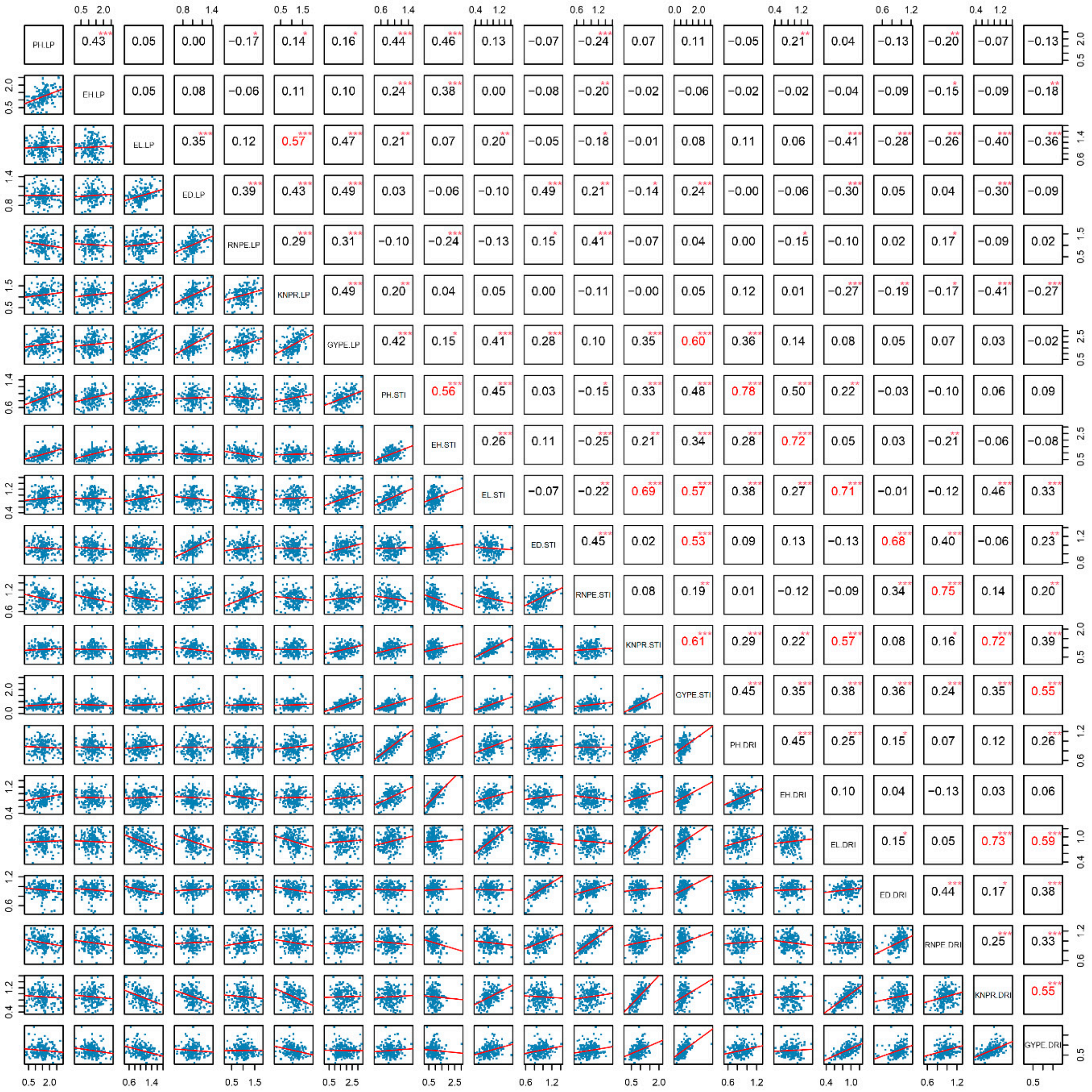

Figure S3. Correlations of stress tolerance indices and phenotypic plasticity of seven agronomic traits. Note: *:$P < 0.05$; **:$P < 0.01$, ***:$P < 0.001$. In the upper right corner, the numbers of red or deep red indicate moderate or high correlation, respectively. The red lines represent the trend of correlation in the lower left corner. PH: plant height, EH: ear height, GYPE: grain yield per ear, EL: ear length, ED: ear diameter, RNPE: row number per ear, KNPR: kernel number per row. LP: Line plasticity of phenotypic plasticity, STI: stress tolerance index, DRI: drought resistance index.

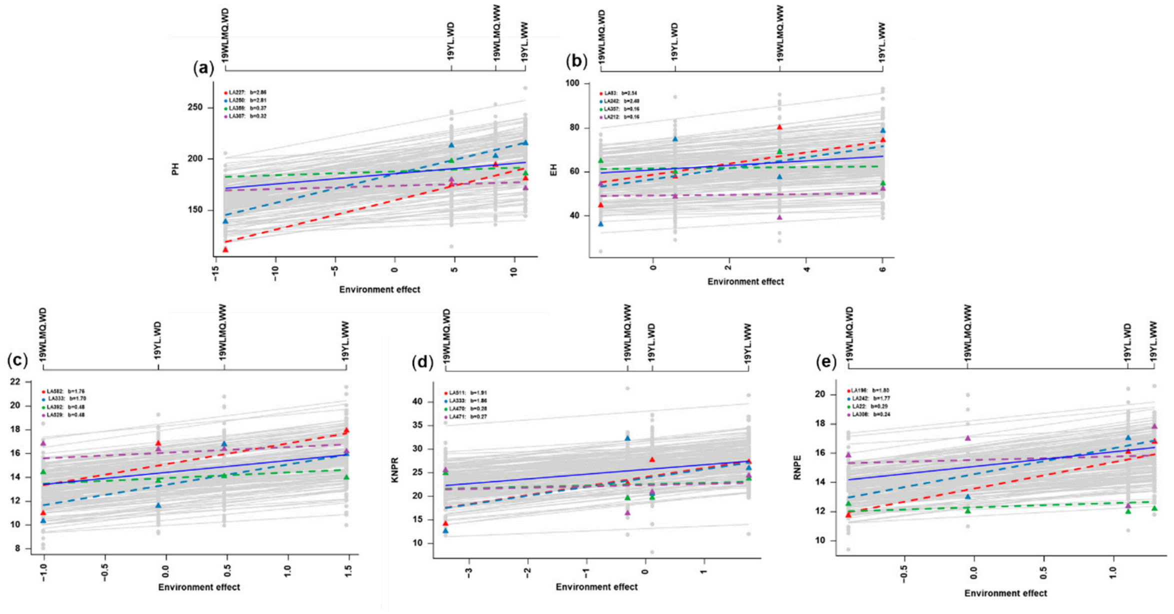

Figure S4. Environment effect and phenotypic plasticity. (**a**) Line plasticity (LP) of plant height (PH), (**b**) LP of ear height (EH), (**c**) LP of ear length (EL), (**d**) LP of kernel number per row (KNPR) (**e**) LP of row number per ear (RNPE). WW: well-watered, WD: water-deficit.

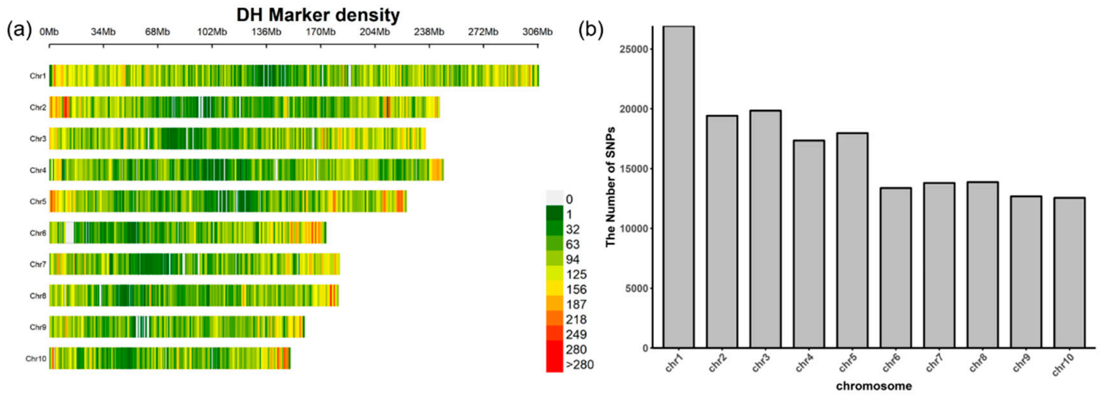

Figure S5. The distribution of single nucleotide polymorphisms (SNPs) on the 10 chromosomes of maize. (**a**) Marker density. (**b**) Number of SNPs per chromosome.

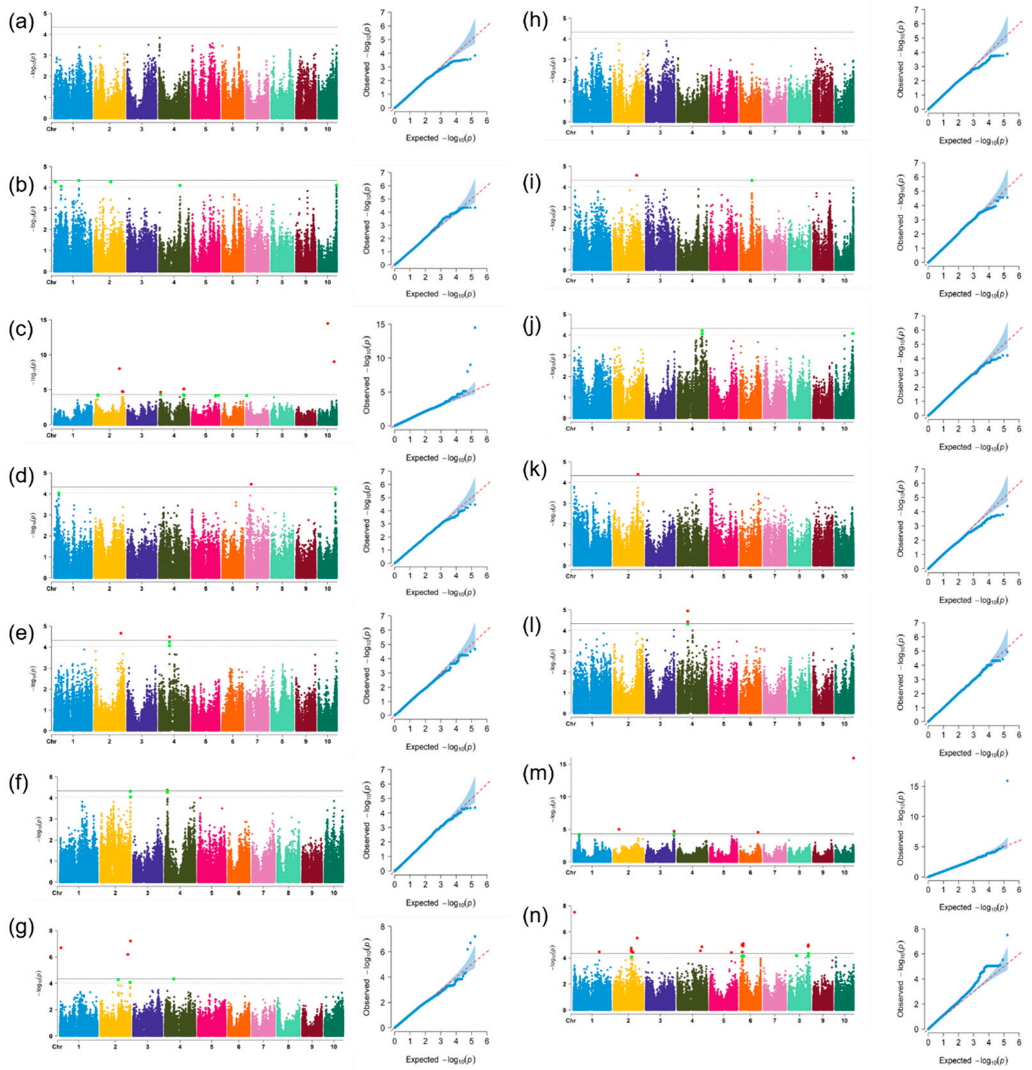

Figure S6. Genome-wide association study of seven agronomic traits under two water conditions. Manhattan plots and QQ plots for seven agronomic traits (**a–g**) under water-deficit (WD) conditions and (**h-n**) under well-watered (WW) conditions. From top to bottom, following order: plant height (PH), ear height (EH), ear length (EL), ear diameter (ED), row number per ear (RNPE), kernel number per row (KNPR), grain yield per ear (GYPE).

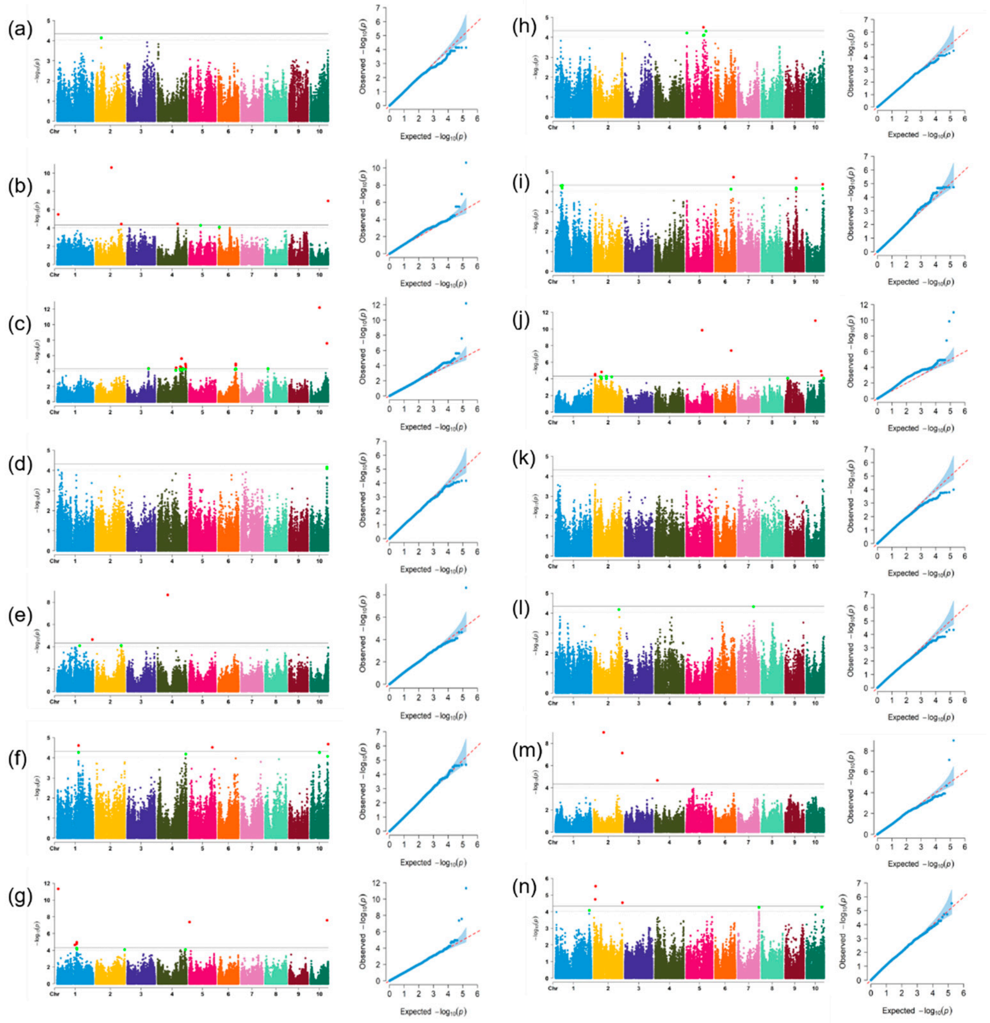

Figure S7. Genome-wide association study of stress tolerance indices of seven agronomic traits. Manhattan plots and QQ plots of seven agronomic traits for (**a–g**) stress tolerance index (STI) and (**h-n**) for drought resistance index (DRI). From top to bottom, following order: plant height (PH), ear height (EH), ear length (EL), ear diameter (ED), row number per ear (RNPE), kernel number per row (KNPR), grain yield per ear (GYPE).

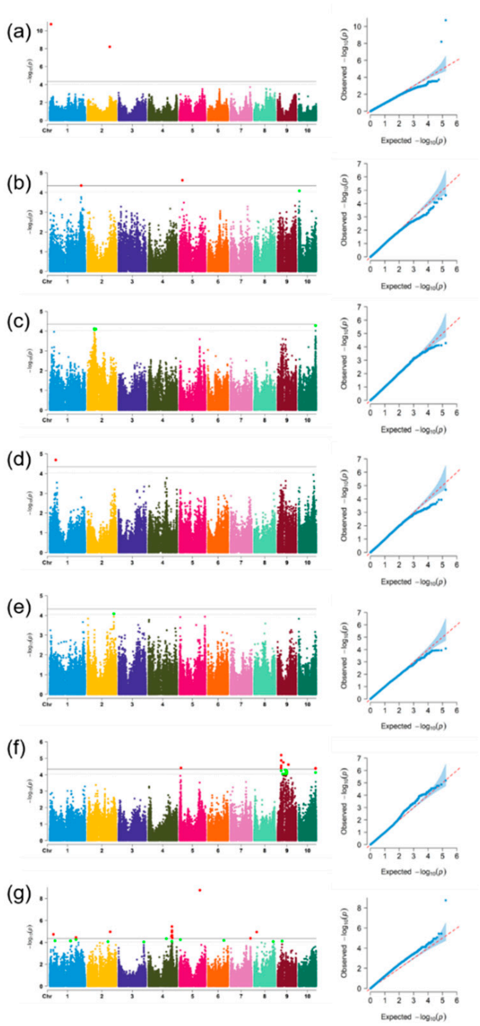

Figure S8. Genome-wide association study of phenotypic plasticity of seven agronomic traits. (**a–g**) Manhattan plots and QQ plots for phenotypic plasticity of seven agronomic traits. From top to bottom, following order: plant height (PH), ear height (EH), ear length (EL), ear diameter (ED), row number per ear (RNPE), kernel number per row (KNPR), grain yield per ear (GYPE).
